# Impact of 3-year changes in lipid parameters and their ratios on incident type 2 diabetes: Tehran lipid and glucose study

**DOI:** 10.1186/s12986-018-0287-6

**Published:** 2018-07-11

**Authors:** Pegah Khaloo, Mitra Hasheminia, Maryam Tohidi, Hengameh Abdi, Mohammad Ali Mansournia, Fereidoun Azizi, Farzad Hadaegh

**Affiliations:** 1 Endocrinology and Metabolism Research Center (EMRC), Vali-Asr Hospital, School of Medicine, Tehran University of Medical Sciences, Tehran, Iran; 2 Prevention of Metabolic Disorders Research Center, Research Institute for Endocrine Sciences, Shahid Beheshti University of Medical Sciences, Number 24, Yemen Street, Shahid Chamran Highway, P.O. Box: 19395-4763, Tehran, Iran; 3 Endocrine Research Center, Research Institute for Endocrine Sciences, Shahid Beheshti University of Medical Sciences, Tehran, Iran; 4 Department of Epidemiology and Biostatistics, School of Public Health, Tehran University of Medical Sciences, Tehran, Iran

**Keywords:** Dyslipidemia, Type 2 diabetes, Triglycerides, LDL-C, HDL-C, Total cholesterol

## Abstract

**Background:**

To examine the impact of changes in all lipid measures including total cholesterol (TC), log-transformed triglycerides (Ln-TG), high density lipoprotein cholesterol (HDL-C), low density lipoprotein cholesterol (LDL-C), non-HDL-C, TC/HDL-C and Ln TG/HDL-C, over an approximate 3 year duration, on incident type 2 diabetes (T2DM).

**Methods:**

A total of 5474 participants, mean age 41.3 years, without prevalent diabetes at baseline or the first follow-up were entered into the study. The association of lipid changes between baseline and the first follow-up i.e., between 1999–2002 and 2002–2005 for those entered in the first phase (*n* = 4406) and between 2002–2005 and 2005–2008 for participants recruited in the second phase (*n* = 1068) with incident T2DM over the follow-up period was assessed, using multivariate Cox proportional hazard analysis.

**Results:**

During a median follow-up of 8.9 years after the second lipid measurements, 577 incident cases of T2DM occurred. After adjustment for a wide variety of confounders and body mass index (BMI) change, each 1-SD increase in TC, Ln-TG, HDL-C, LDL-C, non-HDL-C, Ln-TG/HDL-C and TC/HDL-C was associated with 12, 14, 0.86, 12, 16, 15 and 13% risk for T2DM, respectively (all *p*-values < 0.05). However, after further adjustment for fasting plasma glucose (FPG) change, the risk disappeared for all lipid measures, excluding HDL-C [hazard ratio (HR): 0.84 (0.76–0.93)], Ln-TG/HDL-C [1.14 (1.04–1.25)] and TC/HDL-C [1.12 (1.04–1.21)].

**Conclusions:**

Three year changes in all lipid parameters, after adjustment for known risk factors of T2DM and BMI changes, were associated with incident T2DM. The independent risk of HDL-C and its ratios remained even after adjustment for FPG changes.

**Electronic supplementary material:**

The online version of this article (10.1186/s12986-018-0287-6) contains supplementary material, which is available to authorized users.

## Background

Prevalence of type 2 diabetes (T2DM) is fast increasing as a result of changes in lifestyle, physical inactivity, nutrition transition and a steep increase in obesity [1]. It is assumed that by the year 2045, over 629 million people worldwide will suffer from diabetes, of which over 13% (82 million) will be from the Middle East region [2, 3]. The annual incidence of T2DM is estimated to be over 1% in Iranian population [4]. Numerous studies have discussed the role of potential risk factors including high-risk ethnicity, physical inactivity, obesity, history of cardiovascular disease (CVD), hypertension, gestational diabetes, family history of T2DM, and glucose intolerance in the occurrence of T2DM [5–8]. It has been shown that changes in classic risk factors including body mass index (BMI), blood pressure and fasting plasma glucose (FPG) play a major role in the development of T2DM [9–11].

Many studies have demonstrated the incidence of T2DM to also be associated with elevated triglycerides (TG) and decreased high density lipoprotein cholesterol (HDL-C) levels using only baseline measurements of these lipoprotein measures [12–14]. Lipid ratios can also aid in the prediction of the incidence of T2DM. Some evidence suggests that TG/HDL-C and total cholesterol (TC)/HDL-C are independent risk factors for T2DM [15, 16]. Some data indicate that diabetic dyslipidemia per se is a causal factor for insulin resistance [17]. Increased liver fat leads to hepatic insulin resistance (IR), and the excessive free fatty acids (FFA) derived from circulatory or deposited fat suppress insulin secretion from β cells [18]. Mendelian randomization studies have reported conflicting results regarding the association between low HDL-C and incident T2DM [19, 20].

Similar to other risk factors, some studies indicated that besides values of lipid levels, dynamic changes in TG and HDL-C levels are risk factors of incident T2DM [21, 22]. To the best of our knowledge, however, there is no data regarding the change in other lipid parameters.

This is the first study investigating the impact of changes in TC, low density lipoprotein cholesterol (LDL-C), HDL-C, none-HDL-C, TG/HDL-C and TC/HDL-C on incident T2DM. In the current study, we aimed to increase the present knowledge available on the association of lipid profiles with incident T2DM by examining the impact of changes in levels of all lipid parameters and their ratios, over approximately 3 years, on incident T2DM in adult population of the Tehran Lipid and Glucose study (TLGS).

## Methods

### Study population

Detailed descriptions of the TLGS have been reported elsewhere [23]. Briefly, the TLGS is a community-based prospective study performed on a representative sample of residents of district 13 of Tehran, the capital city of Iran. A total of 15,005 residents were recruited at baseline (1999–2002), and another 3550 residents from the second phase (2002–2005) of study. In the 2nd phase of the TLGS, almost one-third of the participants took part in community-based intervention through community education.

**Fig. 1.**
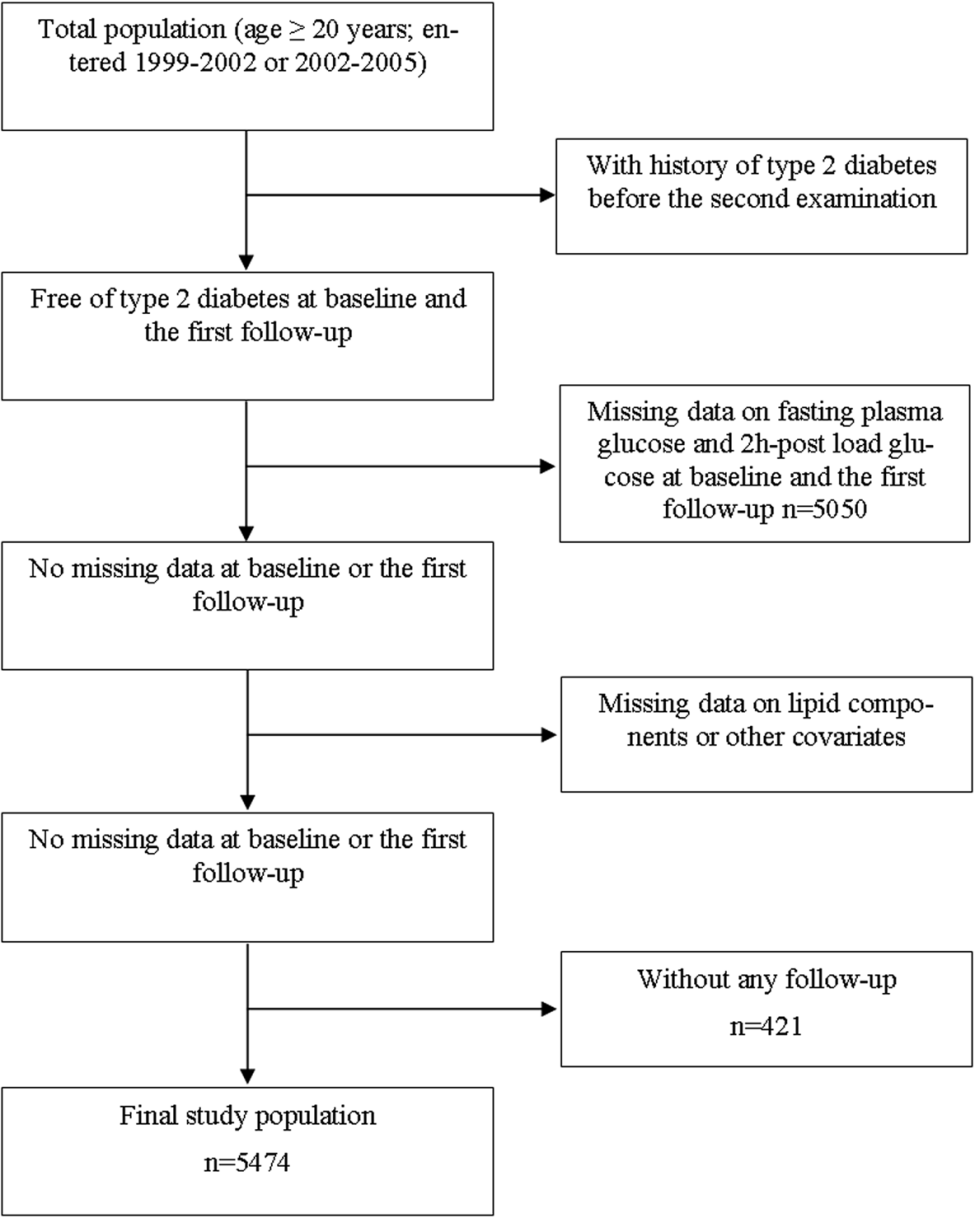
The study population

Interventions were aimed at lifestyle modifıcation by improving nutrition and dietary patterns, increasing physical activity levels and quitting cigarette smoking. Based on TLGS protocol, the whole population was followed at approximately 3 year intervals [(2002–2005), (2005–2008), (2008–2011), (2011–2014)]. Of 18,555 participants, we enrolled 12,808 individuals with age ≥ 20 years in the current study, which evaluated the effects of changes in lipid measures between baseline and the first follow-up i.e., between 1999 and 2002 and 2002–2005 for those entered in the first phase and between 2002 and 2005 and 2005–2008 for participants recruited in the second phase on the incidence of T2DM over the follow-up period; hence, there were 3 and 2 follow-ups for participants who entered in the first and second phases, respectively. Participants with prevalent diabetes at baseline or the first follow up were excluded, leaving 11,191 subjects. After further exclusion of those with missing data on FPG and 2-h post load glucose (2 h-PLG) (*n* = 5050), lipid profile parameters or other covariates (*n* = 246) or those without any follow-up (*n* = 421), 5474 subjects (4406 from the first phase and 1068 from the second phase) remained, who were monitored for a median period of 8.9 years after the second measurement of lipid parameters (Fig. 1).

Medical history, clinical examination and laboratory measurements:

Demographic information, family history of T2DM, history of CVD, medication and current smoking status were obtained from participants during interviews, using a valid questionnaire at the baseline recruitment and each follow-up. Details of anthropometric measurements including weight, height, waist circumference (WC), systolic and diastolic blood pressure (SBP and DBP, respectively) have been previously documented elsewhere [23]. BMI was computed as weight in kilograms divided by height per square meter (kg/m^2^).

After 12 h of fasting, venous blood samples were collected for the biochemical analysis. For all participants, standardized 2 h-PLG test was performed by administrating 75-g anhydrous glucose orally. FPG and 2 h-PLG were measured by an enzymatic colorimetric method using glucose-oxidase. TC was assayed using the enzymatic colorimetric method with cholesterol esterase and cholesterol oxidase. HDL-C was measured after precipitation of the apolipoprotein B-containing lipoproteins with phosphotungstic acid. TG was assayed using glycerol phosphate oxidase. Both inter- and intra-assay CVs were < 1.9, 3 and 2.1% for TC, HDL-C and TG, respectively, in all baseline and follow-up assays. We used a modified Friedewald formula to calculate LDL-C [24]. Analyses were performed using Pars Azmon kits (ParsAzmon, Tehran, Iran) and a Selectra 2 auto-analyzer (Vital Scientifi c, Spankeren, Netherlands). All samples were analyzed only when internal quality control met acceptable criteria.

### Definition of terms

T2DM was considered to be present if the participant was using antidiabetic drugs or if FPG was ≥7 mmol/L or if the 2 h-PLG was ≥11.1 mmol/L [8]. A positive family history of T2DM was defined as having at least one parent or sibling with T2DM. Smoker was defined as occasional or daily user of any amount of cigarette [23] and smoking status was categorized as current vs past or never. We classified education status into three groups: < 6 years, 6–12 years and ≥ 12 years. Hypertension was defined as SBP ≥ 140 mmHg or DBP ≥ 90 mmHg or use of any hypertension drugs.

### Statistical analysis

Mean ± standard deviation (SD) for continuous and frequencies (%) for categorical variables were used to show baseline characteristics of participants. Comparison of baseline characteristics between participants with and without incident T2DM was done using Student’s t-test for continuous variables, Chi-square test for categorical variables and Mann-Whitney test for skewed variables as appropriate. To clarify whether there was any significant clinical difference between respondents and non-respondents (i.e. those with missing data of lipid profile and covariates at the baseline or their first follow-up visit, and those without any follow-up), the mean difference [95% Confidence interval (CI)] of continuous variables and mean differences in the prevalence [95% CI] of each categorical variable were estimated.

Cox proportional hazard regression was used to assess the association between lipid changes with incident T2DM. Event date for incident cases of T2DM was defined as mid-time between the date of follow-up visit at which T2DM was detected for the first time, and the most recent follow-up visit preceding the diagnosis; the follow-up time was drawn from the difference between the calculated mid-time date and the first follow-up. For censored participants, survival time was calculated as the interval between the first and the last observation dates. Follow-up duration was calculated using the measured survival time.

Interaction of lipid changes for all lipid components with gender, was examined in the multivariate model. Since no interaction was found between sex and lipid changes (all *p*-values > 0.05), all analyses were performed in the pooled sample to achieve full statistical power.

Univariable Cox analysis was performed for each potential risk factor including age, sex, family history of T2DM and CVD, education, using lipid or anti-hypertensive drugs, being in intervention group, smoking, physical activity, SBP, DBP, hypertension, as well as change in BMI, WC, WHR and FPG, then, covariates with a *p*-value < 0.2 in the initial univariable analysis were selected to enter the multivariable model [25, 26]. We examined changes in lipid measures (TC, TG, HDL-C, LDL-C, non-HDL-C, TG/HDL-C, TC/HDL-C) as both a continuous and categorical variables. In the categorical model, we categorized the exact amount of change in concentrations (lipid concentration in the first follow-up minus baseline lipid measurement) into tertiles, given the 1st tertile as reference. In the continuous model, we calculated hazard ratio (HR) for each 1 SD change of each lipid measure. For TG and TG/HDL-C, HRs were calculated for 1 SD change in log transformed TG and TG/HDL-C. Four models were defined: Model 1 was adjusted for age, sex, and baseline lipid measurements; Model 2 was further adjusted for educational level, lipid lowering drug, family history of T2DM, history of CVD, hypertension, baseline levels of FPG, BMI and WC; in model 3, BMI change was added to the list of confounders and model 4 included model 3 plus FPG change.

We examined the presence of multi-collinearity by calculating the variance inflation factor (VIF) between baseline measurements of covariates and their changes (lipid measurements and their changes, BMI and its changes and FPG level and its changes) in the regression models. None of the VIFs for the multivariate models exceeded 5, confirming multi-collinearity was unlikely.

We assessed Akaike’s information criteria (AIC) (a statistical estimate of the trade-off between the likelihood of a model against its complexity) as indicators of goodness of fit of the predictive models. A lower value of AIC indicates a better model fit. The discrimination ability of models was calculated using the Harrell’s C statistic.

The proportional hazards assumption in the Cox model was assessed with the Schoenfild residual test indicating all proportionality assumptions were appropriate. Statistical analysis was performed using SPSS for windows version 20 and STATA version 12; *p*-values ≤0.05 were considered statistically significant.

## Results

We included a total of 5474 non-diabetic participants, mean (SD) age of 41.3 (13.6) years, eligible for the study at baseline. The comparison between respondents and non-respondents is shown in Additional file 1: Table S1. No clinically significant differences were observed between respondents and non-respondents.

During a median of 8.9 year follow-up after the second lipid measurements, 577 incident cases of T2DM occurred. Baseline characteristics of subjects with and without incidence of T2DM as well as whole population are shown in Table 1. Individuals with incident T2DM were significantly older and less educated. SBP, DBP, presence of hypertension and CVD, family history of T2DM, lipid drug use, and baseline levels of WC, BMI and FPG were significantly higher in individuals with incident T2DM; however, no differences were found regarding changes in BMI and WC and being in the intervention group. Except for HDL-C which was lower in incident cases of T2DM, all lipid components were higher in this group. Changing values of lipid profiles and their distribution in tertiles including Ln-TG, TC, LDL-C, HDL-C, non-HDL-C, Ln-TG/HDL-C and TC/HDL-C have been summarized in Table 2.

**Table 1:** Baseline characteristics of participants with and without incident T2DM; Tehran Lipid and Glucose Study (TLGS)

	Total (*N* = 5474)	With incident T2DM (*N* = 577)	Without incident T2DM (*N* = 4897)	P-value
*Continuous variables*				
Age (year)	41.3 ± 13.6	46.7 ± 12.7	40.6 ± 13.6	< 0.001
SBP (mmHg)	116.95 ± 17.1	124.2 ± 18.2	116.1 ± 16.8	< 0.001
DBP (mmHg)	76.6 ± 10.4	80.8 ± 10.5	76.1 ± 10.2	< 0.001
Baseline BMI (kg/m^2^)	26.7 ± 4.5	29.1 ± 4.6	26.4 ± 4.4	< 0.001
Baseline WC (cm)	87.6 ± 11.8	94.6 ± 11.1	86.8 ± 11.6	< 0.001
Baseline FPG (mmol/L)	4.95 ± 0.51	5.36 ± 0.60	4.90 ± 0.47	< 0.001
TC (mmol/L)	5.29 ± 1.16	5.74 ± 1.26	5.24 ± 1.13	< 0.001
LnTG	0.44 ± 0.54	0.70 ± 0.52	0.40 ± 0.54	
HDL-C (mmol/L)	1.09 ± 0.28	1.06 ± 0.27	1.09 ± 0.28	0.005
LDL-C (mmol/L)	3.37 ± 0.94	3.68 ± 1.00	3.33 ± 0.92	< 0.001
Non-HDL-C (mmol/L)	4.20 ± 1.16	4.68 ± 1.25	4.15 ± 1.14	< 0.001
LnTG/HDL-C	0.38 ± 0.69	0.68 ± 0.65	0.35 ± 0.69	
TC/HDL-C	5.16 ± 1.68	5.70 ± 1.73	5.09 ± 1.66	< 0.001
BMI change (Kg/m^2^)	0.79 ± 1.97	0.98 ± 2.50	0.77 ± 1.90	0.06
WC change (cm)	3.64 ± 6.79	3.99 ± 6.60	3.59 ± 6.81	0.18
FPG change (mmol/L)	0.03 ± 0.50	0.16 ± 0.57	0.02 ± 0.49	< 0.001
*Categorical variables*				
Male	2320(42.4)	237(41.1)	2083(42.5)	0.53
Hypertension (%)	951 (17.4)	174(30.2)	777(15.9)	< 0.001
Family history of T2DM (%)	1397(25.5)	212(36.7)	1185(24.2)	< 0.001
CVD history (%)	171(3.1)	30(5.2)	141(2.9)	0.005
Education level (%)				< 0.001
≥ 12 years	752(13.7)	51(8.8)	701(14.3)	
6–12 years	3070(56.1)	276(47.8)	2794(57.1)	
< 6 years	1652(30.2)	250(43.3)	1402(28.6)	
Intervention (%)	2425(44.3)	239(41.4)	2186(44.6)	0.14
Smoking (%)				0.74
Never or past	4832(88.4)	513(89.1)	4319(88.3)	
Current	634(11.6)	63(10.9)	571(11.7)	
Lipid drug use (%)	125(2.3)	27(4.7)	98(2.0)	< 0.001

Values are mean ± SD for continuous variables, and n (%) for categorical variables

*T2DM* type 2 diabetes, *SBP* systolic blood pressure, *DBP* diastolic blood pressure, *BMI* body mass index, *WC* waist circumferences, *FPG* fasting plasma glucose, *TC* total cholesterol, *TG* triglyceride, *HDL-C* high density lipoprotein cholesterol, *LDL-C* low density lipoprotein cholesterol, *CVD* cardiovascular diseases

LDL-C values were calculated using the modified Friedewald formula

**Table 2:** Lipid profile of the study population, Tehran Lipid and Glucose Study (1999–2015) (n = 5474)

Lipid parameters	changing value	1^st^tertile changing value	2^nd^tertile changing value	3^rd^tertile changing value
TC (mmol/L)	−0.30 ± 0.78	−1.13 ± 0.54	−0.28 ± 0.17	0.49 ± 0.44
LnTG	− 0.02 ± 0.41	− 0.46 ± 0.23	− 0.02 ± 0.097	0.42 ± 0.24
HDL-C (mmol/L)	−0.07 ± 0.24	− 0.37 ± 0.16	− 0.09 ± 0.07	0.19 ± 0.14
LDL-C (mmol/L)	−0.20 ± 0.63	− 0.87 ± 0.44	− 0.17 ± 0.14	0.44 ± 0.35
Non-HDL-C (mmol/L)	−0.23 ± 0.76	−1.04 ± 0.54	− 0.21 ± 0.16	0.53 ± 0.43
LnTG/HDL-C	0.04 ± 0.51	−0.50 ± 0.27	0.04 ± 0.12	0.59 ± 0.29
TC/HDL-C	0.03 ± 1.35	−1.31 ± 0.98	0.04 ± 0.25	1.36 ± 0.94

Data are shown as mean ± SD

*TC* total cholesterol, *TG* triglycerides, *HDL-C* high density lipoprotein cholesterol, *LDL-C* low density lipoprotein cholesterol

LDL-C values were calculated using the modified Friedewald formula

Results of multivariate Cox proportional hazard analysis for 1-SD change in lipid profiles, adjusted for different potential confounders in different models are given in Table 3. All lipid changes were significantly associated with incident T2DM in models 1 and 2. Accordingly, in model 3 which included a wide variety of different confounders of T2DM as well as BMI change, corresponding risks for TC, Ln-TG, HDL-C, LDL-C, non-HDL-C, Ln-TG/HDL-C and TC/HDL-C were 12, 14, 0.86, 12, 16, 15, and 13%, respectively (all *p*-values < 0.05). However, after further adjustment for FPG change in model 4, this association disappeared excluding for changes in HDL-C [HR (95% CI): 0.84 (0.76–0.93)], Ln-TG/HDL-C [HR (95% CI): 1.14 (1.04–1.25)] and TC/HDL-C [HR (95% CI): 1.12 (1.04–1.21)].

**Table 3:** Multivariate Cox proportional hazard analysis of 1 SD increase in the change of different lipid components and ratios, for incident type 2 diabetes, Tehran Lipid and Glucose Study (1999–2015)

Lipid parameters	Hazard ratio (95% CI)	P-value	AIC	Harrell’s C
TC (mmol/L)				
Model 1	1.11(1.02–1.21)	0.019	9489	0.65
Model 2	1.15 (1.05–1.26)	0.003	9043	0.78
Model 3	1.12(1.02–1.23)	0.018	9025	0.78
Model 4	0.999(0.91–1.10)	0.99	8840	0.81
LnTG				
Model 1	1.20(1.10–1.31)	< 0.001	9399	0.69
Model 2	1.18(1.08–1.29)	< 0.001	9021	0.78
Model 3	1.14(1.03–1.25)	0.008	9003	0.79
Model 4	1.09(0.99–1.20)	0.09	8821	0.81
HDL-C (mmol/L)				
Model 1	0.80(0.72–0.88)	< 0.001	9486	0.65
Model 2	0.85(0.77–0.94)	0.001	9041	0.78
Model 3	0.86(0.78–0.95)	0.003	9021	0.78
Model 4	0.84(0.76–0.93)	0.001	8831	0.81
LDL-C (mmol/L)				
Model 1	1.11(1.02–1.22)	0.016	9499	0.65
Model 2	1.14(1.04–1.25)	0.004	9045	0.78
Model 3	1.12(1.02–1.23)	0.018	9026	0.78
Model 4	1.01(0.92–1.11)	0.81	8842	0.81
Non-HDL-C (mmol/L)				
Model 1	1.16(1.06–1.26)	0.001	9474	0.66
Model 2	1.20(1.09–1.31)	< 0.001	9035	0.78
Model 3	1.16(1.06–1.27)	0.001	9019	0.78
Model 4	1.04(0.95–1.15)	0.38	8839	0.81
LnTG/HDL-C				
Model 1	1.23(1.13–1.35)	< 0.001	9408	0.68
Model 2	1.19(1.09–1.31)	< 0.001	9021	0.78
Model 3	1.15(1.05–1.26)	0.002	9004	0.79
Model 4	1.14(1.04–1.25)	0.007	8822	0.81
TC/HDL-C				
Model 1	1.21(1.13–1.29)	< 0.001	9461	0.66
Model 2	1.15(1.07–1.23)	< 0.001	9036	0.78
Model 3	1.13(1.06–1.22)	0.001	9018	0.78
Model 4	1.12(1.04–1.21)	0.003	8833	0.81

TC: total cholesterol; TG: triglycerides; HDL-C: high density lipoprotein cholesterol, LDL-C: low density lipoprotein cholesterol, CI: confidence interval, AIC: Akaike's information criteria

Model 1: adjusted for age, sex, and baseline measurements of lipid profile

Model 2: adjusted for age sex, education, lipid drug use, family history of type 2 diabetes, history of cardiovascular disease, hypertension and baseline measurements of fasting plasma glucose (FPG), body mass index (BMI), waist circumferences (WC) and lipid profile

Model 3: model 2+ BMI change

Model 4: model 3+ FPG change

We also analyzed lipid change as a categorical variable (Table 4). Significant trends were shown for Ln-TG, non-HDL-C, Ln-TG/HDL-C and TC/HDL-C and in model 3 (all *p*-values < 0.05); however after further adjustment for FPG change, this trend remained only for Ln-TG and Ln-TG/HDL-C. The 3rd tertile of Ln-TG/HDL-C (HR: 1.33, CI 1.08–1.63), TC/HDL-C (HR: 1.27, CI 1.03–1.57) and Ln-TG (HR: 1.22, CI 0.99–1.49, *p* = 0.06) increased the risk of T2DM after full adjustment for covariates, including changes in BMI and FPG (model 4). Regarding non-HDL in models 1, 2 and 3 and for TC and LDL-C in model 2, the 3rd tertile showed a significant risk for T2DM.

**Table 4:** Hazard ratios for predicting type 2 diabetes in different models of tertiles of changes in lipid components, Tehran Lipid and Glucose Study (1999–2015)

Lipid parameters	1^st^tertile changing value	2^nd^tertile changing value	3^rd^tertile changing value	P _trend_	AIC	Harrell’s C
	HR (95%CI)	HR (95%CI)	HR(95%CI)			
TC (mmol/L)						
Model 1	1.00	1.08(0.88–1.33)	1.13 (0.90–1.40)	0.56	9496	0.65
Model 2	1.00	1.11(0.91–1.37)	1.25(1.001–1.56)	0.14	9050	0.78
Model 3	1.00	1.07(0.87–1.32)	1.16 (0.92–1.45)	0.46	9031	0.78
Model 4	1.00	0.97(0.79–1.19)	0.95(0.76–1.18)	0.89	8842	0.81
LnTG						
Model 1	1.00	1.08(0.86–1.36)	1.49(1.22–1.82)	< 0.001	9401	0.69
Model 2	1.00	1.03(0.82–1.30)	1.39(1.13–1.70)	0.002	9022	0.78
Model 3	1.00	0.99(0.78–1.24)	1.29(1.05–1.59)	0.013	9003	0.79
Model 4	1.00	0.92(0.73–1.16)	1.22(0.99–1.49)	0.026	8819	0.81
HDL-C (mmol/L)						
Model 1	1.00	0.85(0.68–1.05)	0.74(0.58–0.95)	0.06	9502	0.65
Model 2	1.00	0.90(0.72–1.12)	0.84(0.66–1.07)	0.35	9051	0.77
Model 3	1.00	0.91(0.73–1.13)	0.86(0.68–1.10)	0.49	9031	0.78
Model 4	1.00	0.87(0.70–1.08)	0.81(0.64–1.04)	0.25	8842	0.81
LDL-C (mmol/L)						
Model 1	1.00	1.13(0.92–1.40)	1.20(0.97–1.49)	0.25	9504	0.65
Model 2	1.00	1.16(0.94–1.43)	1.28(1.03–1.59)	0.08	9050	0.78
Model 3	1.00	1.12(0.91–1.38)	1.21(0.97–1.50)	0.24	9031	0.78
Model 4	1.00	1.03(0.83–1.27)	1.00(0.80–1.25)	0.96	8844	0.81
Non-HDL-C (mmol/L)						
Model 1	1.00	1.02(0.82–1.26)	1.31(1.06–1.62)	0.019	9479	0.66
Model 2	1.00	1.05(0.85–1.29)	1.45(1.17–1.80)	0.001	9039	0.78
Model 3	1.00	1.01(0.82–1.25)	1.35(1.08–1.68)	0.008	9022	0.78
Model 4	1.00	0.94(0.76–1.16)	1.11(0.89–1.38)	0.32	8839	0.81
LnTG/HDL-C						
Model 1	1.00	1.23(0.97–1.55)	1.53(1.25–1.86)	< 0.001	9414	0.68
Model 2	1.00	1.12(0.89–1.42)	1.42(1.16–1.74)	0.002	9025	0.78
Model 3	1.00	1.09(0.86–1.38)	1.33(1.08–1.63)	0.016	9007	0.79
Model 4	1.00	1.12(0.88–1.42)	1.33(1.08–1.63)	0.019	8823	0.81
TC/HDL-C						
Model 1	1.00	1.22(0.98–1.52)	1.47(1.19–1.82)	0.002	9474	0.66
Model 2	1.00	1.12(0.90–1.39)	1.43(1.16–1.77)	0.002	9039	0.78
Model 3	1.00	1.09(0.87–1.36)	1.35(1.09–1.67)	0.013	9022	0.78
Model 4	1.00	1.09(0.87–1.36)	1.27(1.02–1.57)	0.08	8838	0.81

*TC:* total cholesterol, *TG:* triglyceride, *HDL-C:* high density lipoprotein cholesterol, *LDL-C:* low density lipoprotein cholesterol, *HR:* hazard ratio, *CI:* confidence interval, *AIC:*Akaike's information criteria.

Model 1: adjusted for age, sex, and baseline measurements of lipid profile

Model 2: adjusted for age sex, education, lipid drug use, family history of type 2 diabetes, history of cardiovascular disease, hypertension and baseline measurements of fasting plasma glucose (FPG), body mass index (BMI), waist circumferences (WC) and lipid profile

Model 3: model 2+ BMI change

Model 4: model 3+ FPG change

As shown in Tables 3 and 4, according to the model fitness as shown by AIC, the fourth model showed the lowest AIC compared to other models. Furthermore, regarding the discriminatory index of different models, as shown by Harrell’s C, we found better discriminatory index moving from model 1 to 4. Generally, the fourth model had the Harrell’s C ≥ 80% for prediction of incident T2DM.

## Discussion

This is the first study to have examined the impact of changing values in different lipid parameters over approximately 3 years for incident T2DM in a population-based cohort. After adjustment for a wide set of important traditional risk factors of T2DM, including age, sex, education level, family history of T2DM, hypertension, history of CVD, lipid lowering drug use, baseline levels of BMI, WC, FPG and lipid parameters along with BMI and FPG changes, we found that a 1 SD increase in LnTG/HDL-C and TC/HDL-C was associated with an over 12% higher risk and for HDL-C with 16% lower risk for incident T2DM. Interestingly after further adjustment for FPG change, which is in the causal pathway, a 1SD increase in TG/HDL-C and TC/HDL-C was associated with more than 12% higher risk and for HDL-C with 16% lower risk for incident T2DM.

In the current study as well as previous studies conducted among adult Tehranian populations, favorable trends were shown for all lipid parameters [27, 28]. It has been reported that over 30% of Iranian families are now consuming less hydrogenated oil than they did in the past [29] which could possibly explain the favorable lipid trend in the TLGS population during recent years. Moreover, we previously showed that the rate of consumption of lipid lowering medications in non-diabetic TLGS participants increased from 1.6% in 1999–2002 to 6.0% in 2008–2011 [28]. It seems increasing education and awareness are the most probable reasons for decrease in levels of lipid parameters rather than use of lipid lowering drugs. Despite the favorable trend in lipid parameters, prevalences of abnormal lipid profiles are still high; in 2008–2011, among non-diabetic TLGS population, prevalences of high TG, low HDL-C, and high non-HDL-C were 45, 47 and 36%, respectively [28].

The effect of dyslipidemia, which is mostly defined by high TG and low HDL-C levels as well as high TG/HDL-C level, on incident T2DM is well known [12, 15, 30–32]. Among an adult Iranian population, during 6 years of follow-up, we also found that TC/HDL-C and TG/HDL-C, but not TC and non-HDL-C, were independent predictors of incident T2DM [16]. TC/HDL-C was reported to be independently associated with later development of T2DM in non-diabetic Korean adults in a longitudinal analysis [33], while in the Janghorbani, et al. study, no significant association of TG/HDL-C and TC/HDL-C with incident T2DM was seen in a high-risk Iranian population [34]. Sadeghi et al. also indicated hypertriglyceridemia was associated with progression of individuals with pre-diabetes to incident T2DM [35]. Regarding the associations of other lipid parameters with incident T2DM, data are controversial. Some of the prospective epidemiological studies reported null association between LDL-C and incident T2DM [36, 37]; even some clinical trials targeting LDL-C with statin therapy showed low LDL-C concentrations were associated with increased risk of T2DM [38]. On the other hand, in a meta-analysis of case-control studies, it was shown that TC and LDL-C among patients with T2DM were higher than controls, although HDL-C was lower, showing that these lipid parameters can also reflect the risk of T2DM [13]. Limited data are available regarding non-HDL. A recent Chinese cohort study reported that non-HDL-C had better performance than traditional cholesterol indices in predicting T2DM among women [39].

Importantly, all above studies are based on one time point assessment of lipid measures at baseline and did not examine the impact of its dynamic change on incident T2DM. So far only 3 studies have examined impact of lipid changes, focusing only on TG or HDL-C, on incident diabetes [14, 21, 22]. The Tirosh et al. study revealed that 2 assessments of TG levels over approximately 5 years improved the association between TG and T2DM in healthy young Israeli men, independently of changes in BMI, physical activity and eating habits [21]. Skretteberg, et al. also showed that > 25% reduction in TG level results in 56% decrease in T2DM risk, compared to unchanged TG levels in healthy middle-aged Norwegian men; however, similar increase in TG level was not associated with higher risk [22]. In addition, LIFE cohort study conducted in several clinical centers in Scandinavia, United Kingdom and United States on hypertensive patients showed changing values of HDL-C over time reflect risk of T2DM more strongly compared to the baseline values of HDL-C [14]; the three above-mentioned studies examined the association between changes in TG and HDL-C and incident T2DM only as categorical variables. The magnitude of risks across tertiles, in our data analysis, is dependent on the absolute risk in the bottom tertile and on the independent variation of each lipid parameter. As acknowledged by Shai et al. in the Nurses’ Health Study, the association with T2DM of 1 SD increase in each lipid parameter might standardize this variation [40]. Therefore, we hope to add to data of previous studies by examining changes in all lipid parameters, whether as continuous or categorical variables, on incident T2DM. In the present study, using both continuous and categorical forms resulted in consistent findings in the final model with minimal differences regarding HDL-C.

Despite extensive studies, the exact role of lipid disorders in the development of T2DM is still unknown. It was assumed at first that hypertriglyceridemia is the only lipid parameter causing IR, the vicious cycle, emphasizing that hypertriglyceridemia could cause IR while IR and compensatory hyperinsulinemia aggravate hypertriglyceridemia [41]. Later evidence suggests that HDL-C also contributes to this mechanism; significant genetic correlations for various HDL-C measurements and insulin concentrations have been reported [42, 43]. Rutti et al. demonstrated that HDL-C also plays an anti-diabetogenic role by protecting β cells from glucose-induced apoptosis. In contrast, LDL-C inhibits the proliferation of β cells and decrease the maximum insulin secretion [44]. Regarding TC some animal studies showed that elevated serum TC may cause increased cholesterol in pancreatic islets which can significantly affect the glucose stimulated insulin secretion, independent of FFA levels [45, 46].

According to our data analysis, as acknowledged by Li et al., nowadays we cannot restrict the effect of dyslipidemia on incident T2DM to certain parameters [17], which is why we included all lipid parameters in our study. Using the Mendelian randomization study, among a Danish population, it was shown that genetically reduced HDL-C did not associate with increased risk of T2DM [20]. White et al., however, using a similar approach from the Global Lipids Genetics Consortium showed a 1-SD elevation in HDL-C was associated with 17% decrease in risk of T2DM [19]. It has been said that decrease in pancreatic fat is associated with the return of β cell function [47], which again highlights the impact of dynamic changes in lipid profiles on the underlying mechanism of T2DM.

There are some limitations to note in our study. First, though TLGS participants are representative of Iran’s population, further studies need to be conducted to determine if our results can be generalized to other population. Second, since we did not have any data on eating habits, we did not consider this important confounder in our data analysis. Mente et al. recently showed that increased carbohydrate intake is associated with lower LDL-C and HDL-C and also higher TG and TC/HDL-C ratio [48]. Additionally, among Iranians, intake of carbohydrate, especially white rice, that was associated with incident T2DM, is high [49]. Third, because physical activity level was assessed with the lipid research clinic in the first phase of the TLGS and by the modifiable activity questionnaire from the 2nd phase [23], we did not enter this variable in our data analysis, although physical inactivity was not shown as an independent predictor for incident T2DM among Tehranian adults [4]. Fourth, we did not have data of HbA1c for our study population. Since the measurement of HbA1C using high performance liquid chromatography method is an expensive measurement, it was not assessed in this large population-based cohort. Nevertheless, the main strength of our study is its large sample size. This is a population-based cohort conducted on a large sample of Iranians with a long-term follow-up of over a decade. Moreover, it is the first study examining the impact of all lipid components on incident T2DM after adjustment for a wide set of traditional risk factors of T2DM.

## Conclusions

This is the first study to reveal the significant associations of 3-year changes in all lipid parameters with incident T2DM, after adjustment for known risk factors of T2DM and BMI changes. The independent risk of HDL-C and its ratios remained even after adjustment for FPG changes.

Mendelian randomization studies through assessing single nucleotide polymorphism for different lipid measures are needed in the future to shed more light on the exact association between lipid disorders and incident T2DM. Moreover, our results warrant randomized clinical trials to examine the impact of lipid changes, especially HDL-C and its ratios, through changes in lifestyle or use of lipid-lowering medications on incident T2DM.

## Additional file

Additional file 1: **Table S1.** Baseline characteristics in respondents and non-respondents. **Table S2.** Characteristics of participants at baseline and the first follow-up (DOCX 19 kb)

## Abbreviations

2 h-PLG: 2-h post load glucose
AIC: Akaike’s information criteria
BMI: Body mass index
CI: Confidence interval
CVD: Cardiovascular disease
DBP: Diastolic blood pressure
FFA: Free fatty acids
FPG: Fasting plasma glucose
HDL-C: High-density lipoprotein cholesterol
IR: Insulin resistance
LDL-C: Low-density lipoprotein cholesterol
SBP: Systolic blood pressure
T2DM: Type 2 diabetes
TC: Total cholesterol
TG: Triglycerides
TLGS: Tehran Lipid and Glucose study
VIF: Variance inflation factor
WC: Waist circumference
