# Supplementary material for: Impact of 3-year changes in lipid parameters and their ratios on incident type 2 diabetes: Tehran lipid and glucose study
Source: Nutr Metab (Lond). 2018 Jul 11;15:50. doi: 10.1186/s12986-018-0287-6 (PMC6042239; doi:10.1186/s12986-018-0287-6)
Supplement: Supplementary file 1 — Table S1. Baseline characteristics in respondents and non-respondents. Table S2. Characteristics of participants at baseline and the first follow-up (DOCX 19 kb) [file 12986_2018_287_MOESM1_ESM.docx]

Table S1 Baseline characteristics in respondents and non-respondents

|  | | |  | Non-respondents  (N=5717) | Respondents  (N=5474) | Mean/proportion difference (95% CI) |
| --- | --- | --- | --- | --- | --- | --- |
| *Continuous variables* | | |  |  |  |  |
|  | Age (year) | |  | 39.3±15.07 | 41.3±13.6 | -1.98[-2.51-(-1.44)] |
|  | SBP (mmHg) | |  | 116.5±18.01 | 116.95±17.1 | -0.44[-1.10-0.22] |
|  | DBP (mmHg) | |  | 76.2±10.9 | 76.6±10.4 | -0.48[-0.88-(-0.08)] |
|  | Baseline BMI (kg/m^2^) | |  | 26.2±4.9 | 26.7±4.5 | -0.51[-0.69-(-0.33)] |
|  | Baseline WC (cm) | |  | 86.5±12.5 | 87.6±11.8 | -1.10[-1.56-(-0.64)] |
|  | Baseline FPG (mmol/L) | |  | 4.95±0.54 | 4.95±0.51 | 0.006[-0.01-0.03] |
|  | TC (mmol/L) | |  | 5.1±1.16 | 5.3±1.16 | -0.16[-0.20-(-0.11)] |
|  | LnTG | |  | 0.39±0.56 | 0.44±0.54 | -0.04[-0.06-(-0.02)] |
|  | HDL-C (mmol/L) | |  | 1.09±0.29 | 1.09±0.28 | 0.002[-0.01-0.01] |
|  | LDL-C (mmol/L) | |  | 3.24±0.93 | 3.37±0.94 | -0.13[-0.17-(-0.10)] |
|  | Non-HDL-C (mmol/L) | |  | 4.04±1.17 | 4.20±1.16 | -0.16[-0.20-(-0.11)] |
|  | LnTG/HDL-C | |  | 0.34±0.71 | 0.38±0.69 | -0.04[-0.07-(-0.02)] |
|  | TC/HDL-C | |  | 5.02±1.72 | 5.16±1.68 | -0.14[-0.21-(-0.08)] |
|  | BMI change (kg/m^2^) | |  | 0.97±2.25 | 0.82±2.00 | 0.15[-0.01-0.30] |
|  | WC change (cm) | |  | 4.33±7.02 | 3.77±6.88 | 0.56[0.04-1.08] |
|  | FPG change (mmol/L) | |  | 0.06±0.53 | 0.03±0.50 | 0.03[-0.01-0.06] |
| *Categorical variables* | | |  |  |  |  |
|  | Male | |  | 2606(45.6) | 2320(42.4%) | 3.2[1.36-5.04] |
|  | Hypertension (%) | |  | 930(16.3%) | 951(17.4%) | -1.1[-2.49-0.28] |
|  | Family history of T2DM (%) | |  | 1356(23.7%) | 1397(25.5%) | -1.8[-3.39-(-0.205)] |
|  | CVD history (%) | |  | 222(3.9%) | 171(3.1%) | 0.76[0.08-1.43] |
|  | Education level (%) | |  |  |  |  |
|  |  | ≥ 12 years |  | 854(15%) | 752(13.7%) | 1.2[-0.098-2.50] |
|  |  | 6-12 years |  | 3247(56.9%) | 3070(56.1%) | 0.71[-1.12-2.54] |
|  |  | < 6 years |  | 1602(28.1%) | 1652(30.2%) | -2.15[-3.84-(-0.47)] |
|  | Intervention (%) | |  | 2567(44.9%) | 2425(44.3%) | 0.6[-1.24-2.44] |
|  | Smoking (%) | |  |  |  |  |
|  |  | Never or past |  | 4536(82.6%) | 4832(88.4%) | -8.92[-10.28-(-7.58)] |
|  |  | Current |  | 953(17.4%) | 634(11.6%) | 5.09[3.8-6.37] |
|  | Lipid drug use (%) | |  | 117(2.0%) | 125(2.3%) | -0.24[-0.78-0.302] |
| Values are mean±SDfor continuous variables, and n (%) for categorical variables.  SBP: Systolic blood pressure; DBP: Diastolic blood pressure; BMI: Body mass index; WC: Waist circumferences; FPG: Fasting plasmaglucose; TC: total cholesterol; TG: triglyceride; HDL-C: high density lipoprotein cholesterol, LDL-C: low density lipoprotein cholesterol; T2DM: type 2 diabetes; CVD: cardiovascular diseases | | | | | | |

Table S2 Characteristics of participants at baseline and the first follow-up

|  | |  | Baseline | First follow-up |
| --- | --- | --- | --- | --- |
|  | Age (years) |  | 41.3±13.6 | 45.50±13.6 |
|  | SBP (mmHg) |  | 116.95±17.1 | 115.2±17.6 |
|  | DBP (mmHg) |  | 76.6±10.4 | 74.5±10.4 |
|  | BMI (Kg/m^2^) |  | 26.7±4.5 | 27.51±4.6 |
|  | WC (cm) |  | 87.6±11.8 | 91.5±11.9 |
|  | FPG (mmol/L) |  | 4.95±0.51 | 4.98±0.52 |
|  | TC (mmol/L) |  | 5.29±1.16 | 4.99±1.04 |
|  | LnTG |  | 0.44±0.54 | 0.42±0.53 |
|  | HDL-C (mmol/L) |  | 1.09±0.28 | 1.02±0.27 |
|  | LDL-C (mmol/L) |  | 3.37±0.94 | 3.17±0.84 |
|  | Non-HDL-C (mmol/L) |  | 4.20±1.16 | 3.97±1.05 |
|  | LnTG/HDL-C |  | 0.38±0.69 | 0.43±0.68 |
|  | TC/HDL-C |  | 5.16±1.68 | 5.19±1.66 |
| Values are mean±SD.  SBP: systolic blood pressure; DBP: diastolic blood pressure; BMI: body mass index; WC: waist circumferences; FPG: fasting plasma glucose; TC: total cholesterol; TG: triglycerides; HDL-C: high density lipoprotein cholesterol, LDL-C: low density lipoprotein cholesterol. | | | | |
